# Method for accurate experimental determination of singlet and triplet exciton diffusion between thermally activated delayed fluorescence molecules†‡

**DOI:** 10.1039/d0sc05190j

**Published:** 2020-11-16

**Authors:** Marius Jakoby, Shahriar Heidrich, Lorenz Graf von Reventlow, Carl Degitz, Subeesh Madayanad Suresh, Eli Zysman-Colman, Wolfgang Wenzel, Bryce S. Richards, Ian A. Howard

**Affiliations:** Institute of Microstructure Technology, Karlsruhe Institute of Technology Hermann-von-Helmholtz-Platz 1 D-76344 Eggenstein-Leopoldshafen Germany ian.howard@kit.edu; Institute of Nanotechnology Technology, Karlsruhe Institute of Technology Hermann-von-Helmholtz-Platz 1 D-76344 Eggenstein-Leopoldshafen Germany; Light Technology Institute, Karlsruhe Institute of Technology Engesserstrasse 13 D-76131 Karlsruhe Germany; Organic Semiconductor Centre, EaStCHEM School of Chemistry, University of St Andrews St Andrews KY16 9ST UK

## Abstract

Understanding triplet exciton diffusion between organic thermally activated delayed fluorescence (TADF) molecules is a challenge due to the unique cycling between singlet and triplet states in these molecules. Although prompt emission quenching allows the singlet exciton diffusion properties to be determined, analogous analysis of the delayed emission quenching does not yield accurate estimations of the triplet diffusion length (because the diffusion of singlet excitons regenerated after reverse-intersystem crossing needs to be accounted for). Herein, we demonstrate how singlet and triplet diffusion lengths can be accurately determined from accessible experimental data, namely the integral prompt and delayed fluorescence. In the benchmark materials 4CzIPN and 4TCzBN, we show that the singlet diffusion lengths are (9.1 ± 0.2) and (12.8 ± 0.3) nm, whereas the triplet diffusion lengths are negligible, and certainly less than 1.0 and 1.2 nm, respectively. Theory confirms that the lack of overlap between the shielded lowest unoccupied molecular orbitals (LUMOs) hinders triplet motion between TADF chromophores in such molecular architectures. Although this cause for the suppression of triplet motion does not occur in molecular architectures that rely on electron resonance effects (*e.g.* DiKTa), we find that triplet diffusion is still negligible when such molecules are dispersed in a matrix material at a concentration sufficiently low to suppress aggregation. The novel and accurate method of understanding triplet diffusion in TADF molecules will allow accurate physical modeling of OLED emitter layers (especially those based on TADF donors and fluorescent acceptors).

## Abstract 2

A method for measuring triplet diffusion between TADF molecules is presented, and implications of limited triplet diffusion for OLEDs discussed.

## Graphical abstract

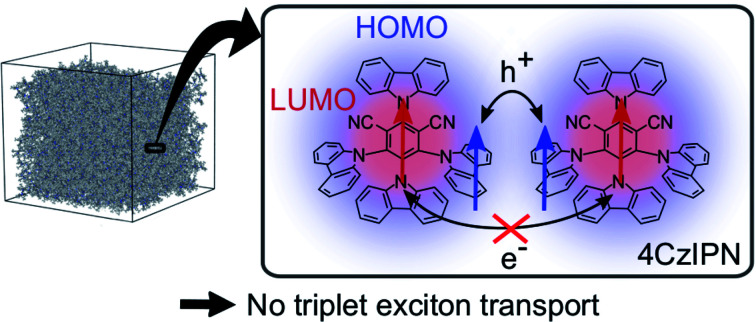

## Introduction

Thermally activated delayed fluorescence (TADF) molecules are typically characterized by a small energy gap (Δ*E*_ST_) between the lowest-excited singlet and triplet excitonic states. Consequently, triplets can convert to singlets by reverse inter-system crossing (RISC).^1^ Such harvesting of the triplet states for emission significantly enhances the efficiency of organic light-emitting diodes (OLEDs) with red, green and blue organic TADF-based OLEDs having been realized with external quantum efficiencies exceeding 25%.^2–4^ In order to provide accurate models and simulations of device physics (especially in devices that utilize TADF dopants and fluorescent acceptors), the diffusion constants for singlets and triplets must be known. However, despite pioneering studies,^5,6^ unambiguous access to triplet diffusion lengths in these materials is lacking. The challenge in determining the diffusion lengths arises in these molecules from the excited-state population cycling multiple times through singlet and triplet states. Menke *et al.* have proposed a method to split the total excited-state quenching (by a quenching layer in a bilayer structure) into contributions from singlet and triplet diffusion based on a theoretical calculation of the Förster energy transfer (FRET) rate to establish the singlet contribution.^5^ In the context of our present results, we conclude that this FRET contribution was underestimated, leading to an overestimation of the triplet diffusion. Yurash *et al.* estimated the change in the rate of delayed fluorescence to be solely due to triplet quenching;^6^ such an estimate (see Section S4‡) would also lead to overestimation of triplet diffusion length.

## Results and discussion

Herein, we present a novel method for accurately determining singlet and triplet diffusion lengths and constants based on easily accessible experimental data. This approach overcomes previous limitations on exciton diffusion in organic TADF molecules (2,4,5,6-tetra(9*H*-carbazol-9-yl)isophthalonitrile (4CzIPN)). The current work provides an accurate and experimentally accessible method to measure triplet diffusion between TADF molecules, demonstrated here using 4CzIPN, 2,3,5,6-tetrakis(3,6-di-*tert*-butyl-9*H*-carbazol-9-yl)benzonitrile (4TCzBN) and quinolino[3,2,1-de]acridine-5,9-dione (DiKTa). Our results on these systems, supported by theoretical calculations, provide the novel insight that triplet diffusion in TADF molecules can be almost negligible. Such limited triplet diffusion is highly favorable for device architectures utilizing fluorescent acceptors alongside TADF donors, and is a likely explanation for the recently observed high efficiencies in such systems.^7–10^

To begin, we derive the key result of this communication, eqn (9) and (11), that allows for the accurate determination of the triplet diffusion length based on time-resolved photoluminescence (PL) measurements of a series of samples with varying quencher concentrations. We start with the well-known coupled rate equations from whose solution the singlet, *c*_s_, and triplet, *c*_t_, excited-state concentrations as a function of time can be found:1
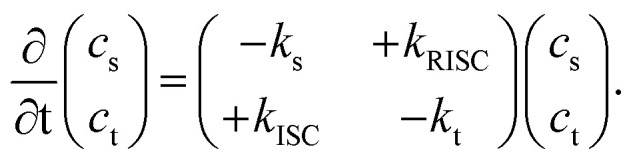
Here, *k*_s_ and *k*_t_, the singlet and triplet decay rates, are given by:

2 *k*_s/t_([*Q*]) = *k*^r^_s/t_ + *k*^nr^_s/t_ + *k*^*Q*^_s/t_[*Q*] + *k*_ISC/RISC_.

These expressions differ from the standard formulation only in the additional term *k*^*Q*^_s/t_[*Q*], which, as in the standard Stern–Volmer (SV) analysis, represents a quenching rate that scales linearly with the concentration of a quenching molecule distributed randomly through the film, [*Q*]. The prefactor *k*^*Q*^_s/t_ is related to the singlet/triplet diffusion constant and length. The diffusion lengths can be extracted through a SV analysis, wherein the SV constant *K*_s/t_[*Q*] is defined by:3
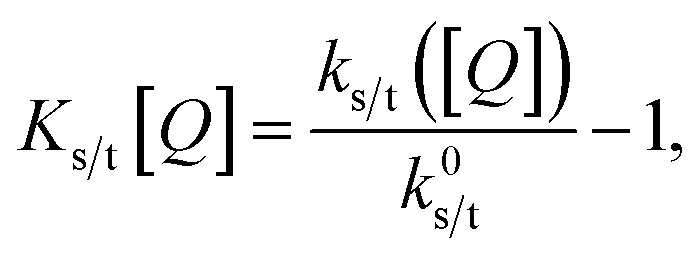
where *k*^0^_s/t_ is *k*_s/t_([*Q*]) when *Q* = 0 (the unquenched singlet or triplet decay rate). Employing the Smoluchowski equation, the following relation between the SV constant *K*_s/t_ and the diffusion length for the singlet or triplet excitons *l*_s/t_ can be derived:^11^4
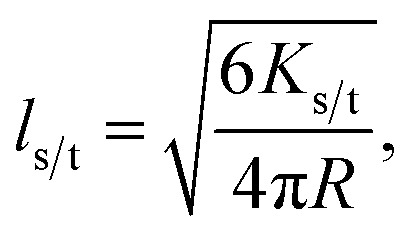
where *R* is the interaction radius that was chosen to be 1 nm for the used quencher TADF molecule combinations.^6^

The commonly used approximation for organic TADF materials that *k*_s_ ≈ *k*_P_ (where *k*_P_ is the measured rate of the prompt fluorescence decay) holds for most materials in this class and we will use it here (as *k*_RISC_ ≪ *k*_s_ is almost always true).^12^ Then, the SV constant for singlets (and therefore the singlet diffusion length during a single pass through the singlet excitonic state) can be easily experimentally found by measuring the quenching of the prompt luminescence as a function of quencher concentration *via* the relationship:5
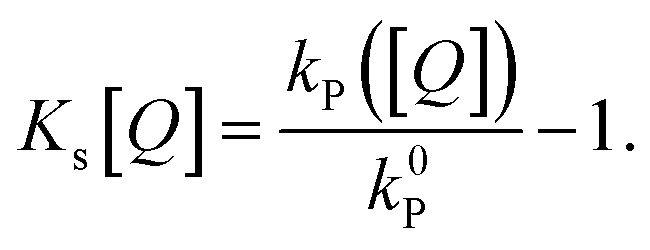


The SV constant for the triplets, *K*_t_[*Q*], is given by:6
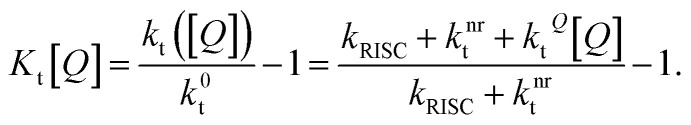


In this form, it is not immediately obvious how *K*_t_[*Q*] should be easily and accurately found from experimental data. The triplet decay rate *k*_t_([*Q*]) (sum of the non-radiative, RISC and quenching rate) is not directly accessible by any optical experiment. However, we show in the following how *K*_t_[*Q*] can be re-expressed in terms of easily observable experimental quantities.

Using the reverse and inter-system crossing efficiencies *ϕ*_RISC_ and *ϕ*_ISC_, respectively, the total PL quantum yield (PLQY) of a TADF molecule, *η*_total_, can be expressed as:7
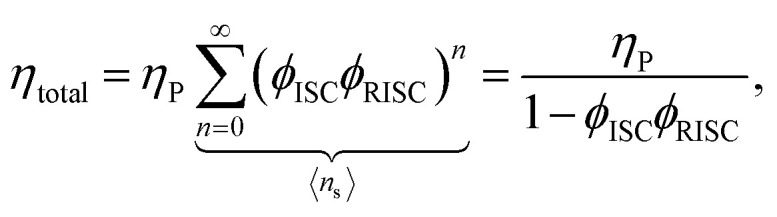
with the prompt PLQY *η*_P_ and the average number of passes through the singlet state 〈*n*_s_〉. Solving for *k*_t_ (using *ϕ*_ISC/RISC_ = *k*_ISC/RISC_/*k*_s/t_) leads to:8
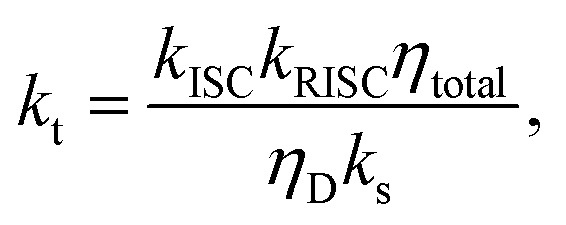
with the delayed PLQY *η*_D_ = *η*_total_ − *η*_P_. Inserting eqn (8) in eqn (6) and again using *k*_s_ ≈ *k*_P_ yields:9
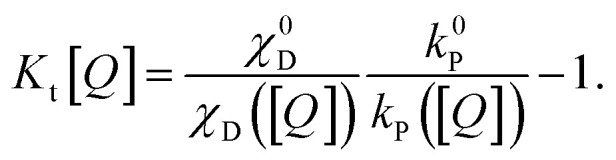
Here, *χ*_D_ = *η*_D_/*η*_total_ is the fraction of delayed over the overall emission. All quantities of eqn (9) can now be measured easily using time-resolved spectroscopy methods and the triplet diffusion length in a single pass through the triplet state can be established.

**Fig. 1:**
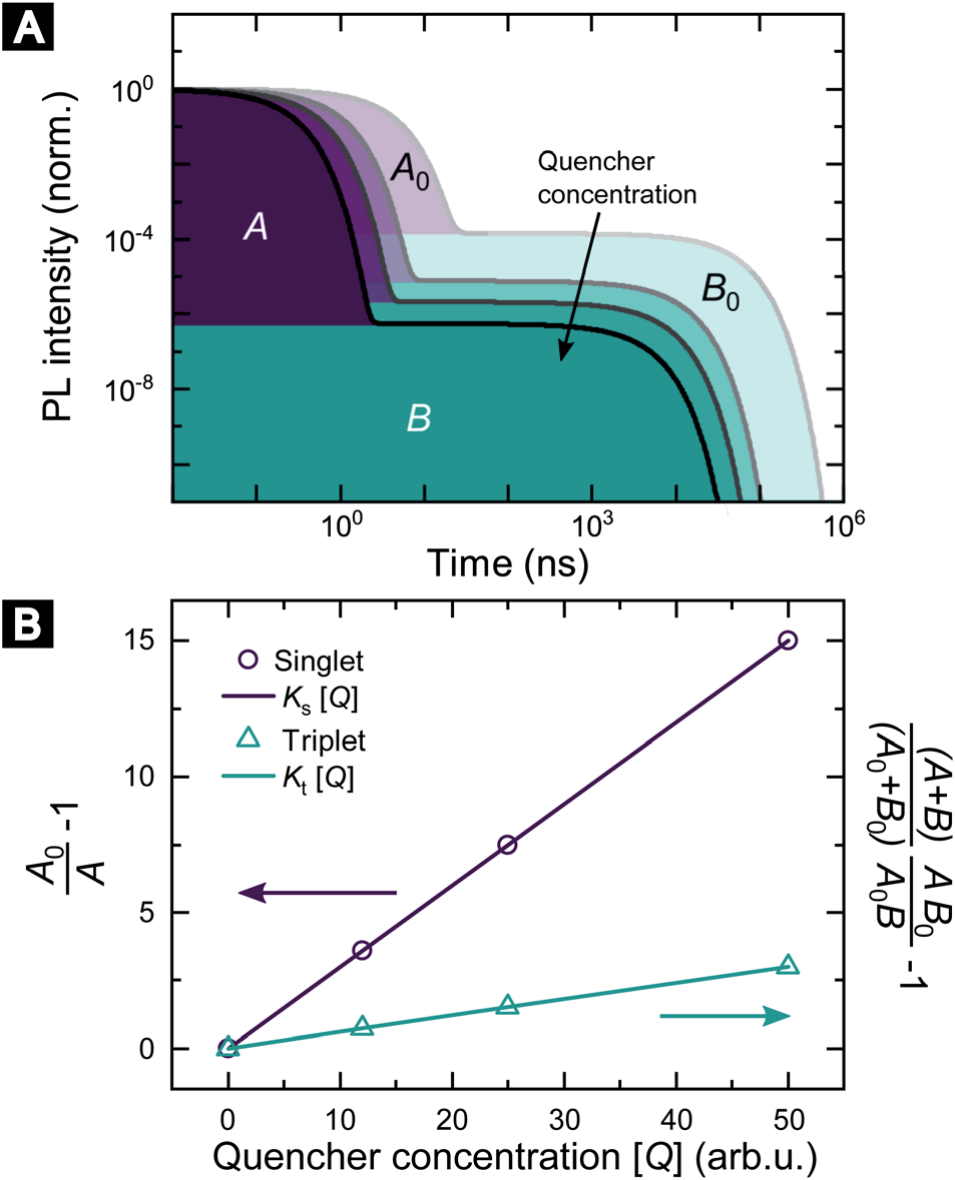
Methodology of determining singlet and triplet diffusion length between TADF molecules. (A) Simulated PL kinetics based on eqn (1) for four different quencher concentrations. (B) SV plot for singlet and triplet excitons based on eqn (10) and (11) using the indicated areas shown in (A).

Fig. 1 illustrates how time-resolved PL data as a function of quencher concentration should be analyzed to determine both the singlet and triplet diffusion lengths, based on eqn (3) and (9). In Fig. 1, the PL intensity curves are simulated with eqn (1) for *k*^*Q*^_s_ = 5*k*^0^_s_[*Q*] and *k*^*Q*^_t_ = *k*^0^_t_[*Q*], *i.e. K*_s_/*K*_t_ = 5 (a complete set of the simulated data and other ratios *K*_s_/*K*_t_ are shown in Fig. S3 and S4‡). Firstly, the data is normalized. Given the monomolecular decays, this allows the delayed and prompt lifetimes to also be found by the integral areas under the respective portions of the curve (labeled A and B). In this case we can rewrite eqn (3) as:10
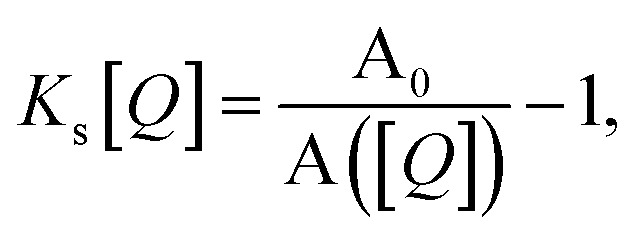
and eqn (9) as:11
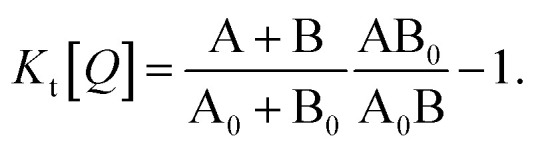


Eqn (10) and (11) allow the singlet and triplet diffusion lengths (during a single pass through the respective state) to be easily determined through two integrals. The total diffusion length (considering all passes through the respective state) and the diffusion constant for the state can then also be easily found by multiplying the single pass diffusion length by the square root of the number of passes through the state, as discussed below in the experimental demonstration. As a side note, in case the prompt PL transient is not monoexponential, *e.g.* due to inhomogeneities in the film, an average decay rate has to be determined and eqn (9) has to be considered.

We demonstrate this method using three benchmark TADF molecules. As PL quenchers we use the electron accepting molecules indene-C60 bisadduct (ICBA) or [6,6]-phenyl-C61-butyric acid methyl ester (PCBM) that act as an electron acceptor and therefore will quench both singlet and triplet excitons with similar transfer rates. We have used an intensified charge-coupled device (ICCD) and/or a streak camera to measure the PL kinetics of the three sample sets. The organic TADF molecules were dispersed in a host matrix of 1,3-bis(*N*-carbazolyl)benzene (mCP) to prevent aggregation of the molecules. For 4CzIPN and 4TCzBN we used a guest concentration of 20 wt%, which is on the high side for the active layer of OLEDs involving those molecules,^13^ in order to determine an upper bound of the triplet diffusion length in these devices. However, due to the tendency of DiKTa to aggregate at mCP concentration of 80% we have lowered the guest concentration to 1 wt% for the DiKTa thin films.

**Fig. 2:**
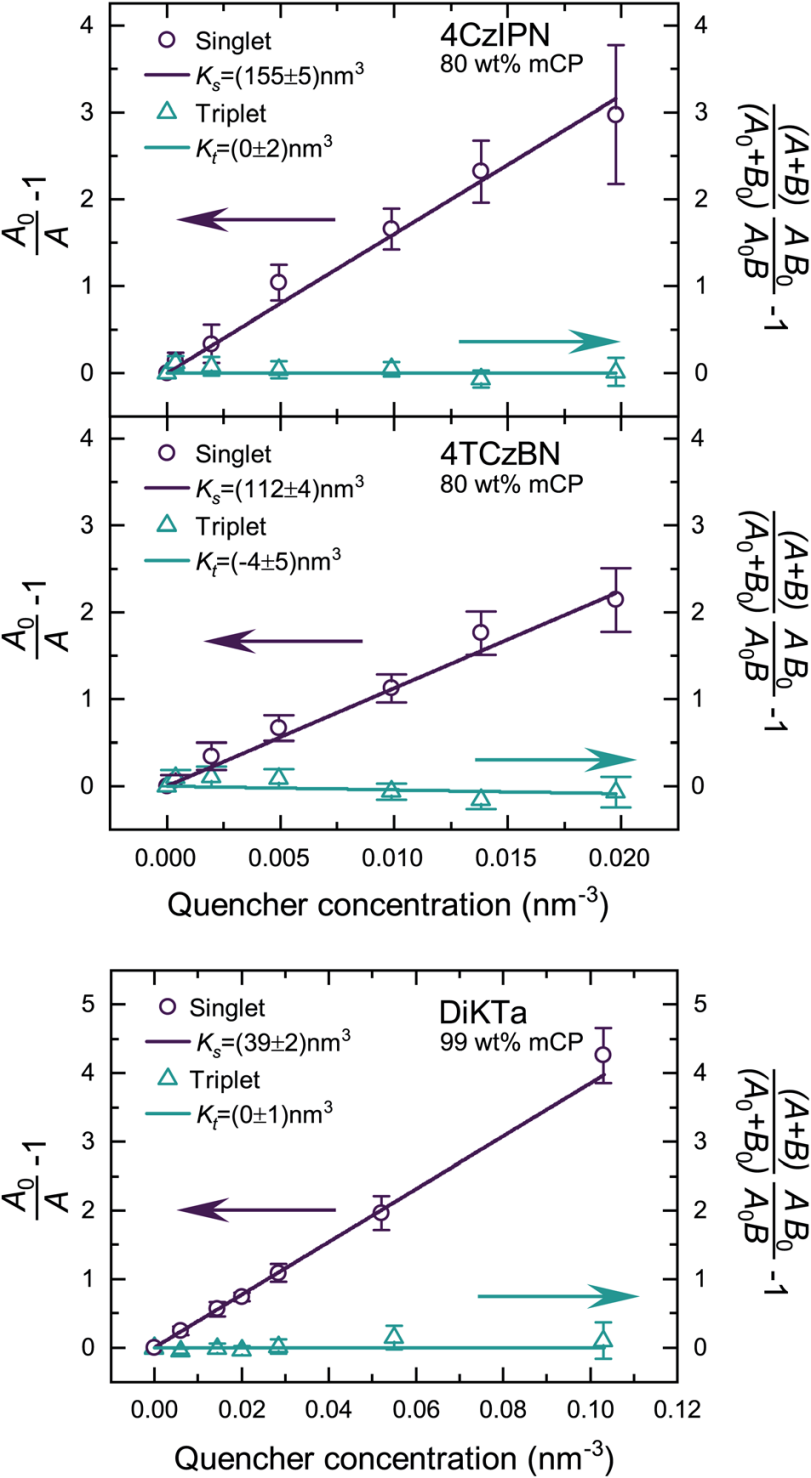
SV plots of the TADF molecules 4CzIPN, 4TCzBN and DiKTa. Molecular structures are displayed in Fig. S1.‡

Fig. S5 and S6‡ show the raw data for the time-resolved PL measurements of the TADF molecules with various quencher concentrations. The data were analyzed according to eqn (10) and (11). Since *k*_P_ ≪ *k*_D_ for the studied molecules, we have approximated area A and B by integration over the prompt and delayed fraction of the PL kinetic, respectively. In Fig. 2 the extracted SV plots are shown and a summary of the resulting diffusion lengths *l*_D_ and diffusion constants is given in Table 1. It should be noted that the above analysis leads to the extraction of the diffusion length during one cycle through the respective exciton. That is, the diffusion lengths within the lifetimes 1/*k*^0^_s_ and 1/*k*^0^_t_ for singlet and triplet excitons, respectively. To determine the total length that the excited-state moves during all cycles through the singlet/triplet state, the diffusion length for a single pass in a state has to be multiplied by the square root of the average number of passes through the state. The number of passes through a state (given the excited-state enters that state once) is given by 〈*n*^0^_s_〉 = (A_0_ + B_0_)/A_0_.

**Table 1:** Singlet and triplet diffusion length and constant for a single cycle and the total number of cycles through the respective state within the lifetime of the exciton. The data is extracted from the linear fits shown in Fig. 2. The determined diffusion parameters correspond to films in which the TADF molecules are dispersed in a mCP host matrix (20 wt% for 4CzIPN and 4TCzBN and 1 wt% for DiKTa)

	Singlet excitons			Triplet excitons		
	Single	Total	*D*_s_ (cm^2^ s^−1^)	Single	Total	*D*_t_ (cm^2^ s^−1^)
	*l*_D_ (nm)	*l*_D_ (nm)		*l*_D_ (nm)	*l*_D_ (nm)	
4CzIPN	8.6 ± 0.2	9.1 ± 0.2	(1.0 ± 0.1) × 10^−5^	<1.0	<1.2	<6 × 10^−10^
4TCzBN	7.3 ± 0.2	12.8 ± 0.3	(2.9 ± 0.2) × 10^−5^	<0.7	<1.0	<8 × 10^−10^
DiKTa	4.3 ± 0.2	8.3 ± 0.3	(7.3 ± 0.5) × 10^−6^	<0.7	<1.3	<2 × 10^−11^

The determined 4CzIPN (in mCP) singlet diffusion length of (9.1 ± 0.2) nm agrees well with the singlet diffusion length determined by Yurash *et al.* in a neat film of 4CzIPN, but there is a substantial difference for the triplet diffusion length.^6^ While Yurash and coworkers stated a triplet diffusion length of 2.8 nm we determined a triplet diffusion length below 1.2 nm (limited by our measurement accuracy). The smaller distance between 4CzIPN molecules in the neat film could increase the exchange coupling and the triplet diffusion length, explaining the difference with our results (which are of more relevance to a device situation wherein the TADF molecule is dispersed in a host). Another contribution may be a slight overestimate by Yurash *et al.* in, to our understanding, basing their triplet quenching efficiencies on the raw change in delayed emission lifetimes.^6^ For all studied molecules a triplet diffusion length of 0 nm is well within the error bounds of the experimental data. The upper limits in Table 1 are determined by using the upper bound of the deviation from the mean value of *K*_t_. However, as will be shown in the following section a triplet diffusion length of essentially 0 nm is the result of our density functional theory (DFT) simulation. As a side note, the slightly negative mean of *K*_t_ for 4TCzBN could be the result of relaxation processes leading to a small overestimation of the singlet exciton quenching within the delayed lifetime. The result of a vanishing triplet diffusion length for 4CzIPN are also supported by a low temperature triplet–triplet annihilation (TTA) study by Niwa *et al.*^14^ Based on the TTA coefficient they determined a triplet diffusion constant of only about 1 × 10^−13^ cm^2^ s^−1^ for 10 wt% 4CzIPN in mCP (at 6 K).^14^

**Fig. 3:**
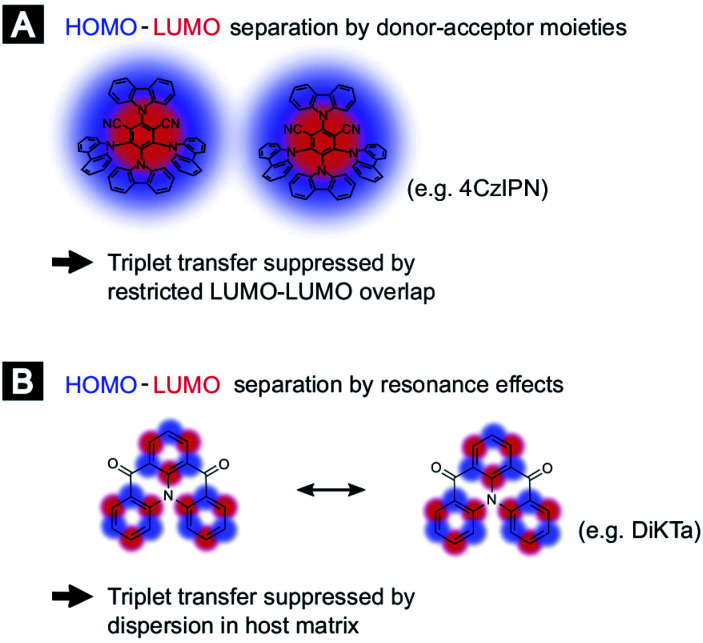
Schematic of exciton motion in TADF based devices. (A) Suppressed triplet transfer as a result of the molecular architecture restricting the LUMO–LUMO overlap for two donor–acceptor type molecules like 4CzIPN. (B) Suppressed triplet transfer due to a highly dispersion (to prevent aggregation) for electron resonance based TADF molecules.

In order to compare the experimental results with theory, we employed DFT calculations to extract Dexter transfer rates based on Marcus theory. Details on these simulations can be found in the ESI (Section S8).‡ Here, we have restricted ourselves to the two molecules 4TCzBN and DiKTa due to the structural similarity of 4TCzBN and 4CzIPN. Fig. S8‡ depicts the determined Dexter transfer rates as a function of distance between the interacting molecules for a film that includes only TADF molecules. The highest occurring rate in the simulation for 4TCzBN was 267 s^−1^ with a center-of-mass distance between the interacting molecules of 1.4 nm. Assuming that between each neighboring molecule this transfer rate would apply, an upper bound of the triplet diffusion constant can be determined to ∼1 × 10^−12^ cm^2^ s^−1^. For the chosen 20 wt% concentration that is also used in devices, the average distance between molecules increases to about 2 nm. The calculated charge-transfer rates reveal that electron transfer is 3 to 4 orders of magnitude lower than the transfer of the hole (compare Fig. S10(B)‡), suggesting the restricted LUMO–LUMO overlap is responsible for the negligible triplet transport in these molecules. Whereas the triplet transport for DiKTa can be significantly faster at short center of mass spacing, at spacings similar to those found in devices the triplet transport is equally low. The mechanisms for the limited triplet transport in these molecules are schematically illustrated in Fig. 3.

The negligible triplet diffusion lengths for donor–acceptor TADF molecules like 4CzIPN and 4TCzBN are supported by recent results of Franco *et al.* where attaching a phenylene ethynylene oligomer to a 4CzIPN core did not lead to quenching of the TADF charge-transfer triplet state by the lower-lying triplet state on the oligomer.^15^ Along this line, a recent comparison of experimental device characteristics and kinetic Monte Carlo simulations came to the conclusion that in a TADF donor-fluorescent acceptor device based on 4CzIPN-Me triplet transport to fluorescent dopants is a negligible loss channel.^16^ Furthermore, Narushima *et al.* recently showed in the context of conjugated molecular crystals that even though the stacked molecules have a good LUMO–LUMO overlap the very small overlap between adjacent highest occupied molecular orbitals (HOMOs) leads to a very small diffusion constant for triplet excitons (3 × 10^−9^ cm^2^ s^−1^).^17^

## Conclusions

In conclusion, we provide an easy and accurate method of measuring triplet diffusion in TADF molecules. This method overcomes the unique challenge posed in TADF systems of the delayed fluorescence lifetime being quenched both by the motion of triplets, and regenerated singlets. We establish that the triplet motion can be negligible compared to the singlet motion in TADF molecules, a situation that is highly favorable for device applications requiring transfer of singlets to a fluorescence acceptor.^18^ Given this method relies only on standard experimental data, we anticipate that it will be of broad use for increasing the fundamental understanding of TADF materials, and obtaining accurate parameters to allow physical modeling of device performance.

## Conflicts of interest

There are no conflicts to declare.

## Supplementary Material

SC-012-D0SC05190J-s001
