# Supplementary material for: Method for accurate experimental determination of singlet and triplet exciton diffusion between thermally activated delayed fluorescence molecules
Source: Chem Sci. 2020 Nov 16;12(3):1121–5. doi: 10.1039/d0sc05190j (PMC8179038; doi:10.1039/d0sc05190j)
Supplement: SC-012-D0SC05190J-s001 [file SC-012-D0SC05190J-s001.pdf]

Electronic Supplementary Material (ESI) for Chemical Science.
This journal is © The Royal Society of Chemistry 2020

Supporting Information

Method for Accurate Experimental Determination of Singlet and Triplet Exciton Diffusion between Thermally Activated Delayed Fluorescence Molecules

Marius Jakoby,[a] Shahriar Heidrich,[b] Lorenz Graf von Reventlow,[c] Carl Degitz,[b] Subeesh Madayanad Suresh,[d] Eli Zysman-Colman,[d] Wolfgang Wenzel,[b] Bryce S. Richards,[ac] and Ian A. Howard*[ac]

S1. Experimental

Time-resolved PL measurements were performed using a gated camera setup or a streak camera setup. For the ICCD measurements, as excitation we used the third harmonic of an diode pumped solid state (DPSS) Nd:YLF laser (picolo1, Innolas GmbH), emitting pulses of 0.8 ns pulse duration and a wavelength of 355 nm. The PL emission was detected by a spectrograph (Acton SpectraPro-2300, Princeton Instruments Inc.) coupled to an ICCD camera (PiMax 4, Princeton Instruments Inc.). For the streak camera measurements, we used the third harmonic (343 nm of a mode-locked ytterbium laser (Light Conversion, Pharos) with a pulse width of 190 fs as an excitation source. For the data acquisition we used a universal streak camera (C10910-02, Hamamatsu) coupled to a spectrometer (Kymera 328i, Andor). During the measurements, the samples were kept under dynamic vacuum with pressures below 1×10^{-5} hPa. All PL transients were collected at a fluence of $330\,\mathrm{nJ\,cm^{-2}}$.

S2. Sample preparation

The samples were prepared from solution and spin coated on quartz substrates. Tetrahydrofuran (THF) was used as a solvent for 2,4,5,6-tetra(*9H*-carbazol-9-yl)isophthalonitrile (4CzIPN) and 2,3,5,6-tetrakis(3,6-di-*tert*-butyl-9*H*-carbazol-9-yl)benzonitrile (4TCzBN) samples, and dichloromethane (DCM) as a solvent for quinolino[3,2,1-de]acridine-5,9-dione (DiKTa) samples. Solutions were prepared using a concentration of 3 g/L. For each TADF molecule 7 samples with different quencher (weight) concentration were spin coated. As a quencher indene-C60 bisadduct(ICBA) was used for 4CzIPN and 4TCzBN, and [6,6]-Phenyl-C61-butyric acid methyl ester (PCBM) as a quencher for DiKTa. 4CzIPN and 4TCzBN were dispersed in 1,3-bis(*N*-carbazolyl)benzene (mCP) with a weight concentration of 20 % while DiKTa at a concentration of 1 %.

S3. Quencher concentrations

To determine the quencher concentration in units of $1\,\mathrm{nm^{-3}}$ the weight and film thicknesses were determined using a quartz microbalance and white-light interferometry, respectively, leading to an average film density of $1.25\,\mathrm{g\,cm^{-3}}$ of evaporated films of mCP:4TCzBN (80:20 wt/wt). We assume the density of the spin coated films to be comparable. As we only changed the emitters, we assumed similar densities for mCP:4CzIPN and mCP:DiKTa thin films. Using the molecular weight of ICBA ($952.96\,\mathrm{g\,mol^{-1}}$), PCBM ($911\,\mathrm{g\,mol^{-1}}$), 4CzIPN ($788.89\,\mathrm{g\,mol^{-1}}$), 4TCzBN ($1212.73\,\mathrm{g\,mol^{-1}}$), DiKTa ($297.31\,\mathrm{g\,mol^{-1}}$) and mCP ($408.49\,\mathrm{g\,mol^{-1}}$) the weight percentages of the quencher were translated to quencher concentrations inside the films.

[a] *Institute of Microstructure Technology, Karlsruhe Institute of Technology, Hermann-von-Helmholtz-Platz 1, D-76344 Eggenstein-Leopoldshafen, Germany. E-mail: ian.howard@kit.edu*
[b] *Institute of Nanotechnology Technology, Karlsruhe Institute of Technology, Hermann-von-Helmholtz-Platz 1, D-76344 Eggenstein-Leopoldshafen, Germany.*
[c] *Light Technology Institute, Karlsruhe Institute of Technology, Engesserstrasse 13, D-76131 Karlsruhe, Germany.*
[d] *Organic Semiconductor Centre, EaStCHEM School of Chemistry, University of St Andrews, St Andrews KY16 9ST,UK.*

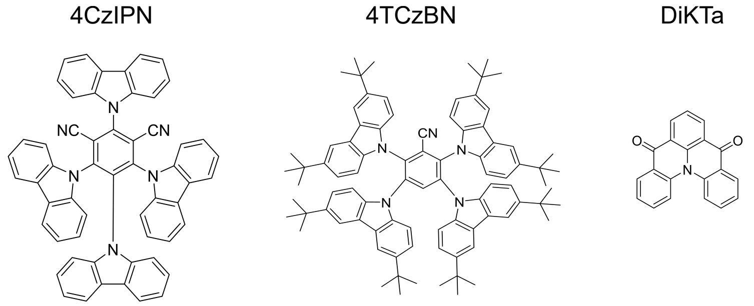

Fig. S1 Chemical structures of the three studied TADF molecules.

S4. Stern Volmer Analysis based on delayed decay rate

In principle, it is also possible to determine the triplet diffusion length by measuring the prompt and delayed decay rate. However, in this case the ISC and RISC rates would be additionally needed (*vide infra*), which makes this approach poorly applicable in practice. Furthermore, it should be noted that the quenching of the delayed decay rate is not directly correlated to the triplet diffusion length.

The delayed lifetime is given by:

$$k_\mathrm{D} = 1/2(k_\mathrm{s} + k_\mathrm{t} - \{k_\mathrm{s}^2 - 2k_\mathrm{s}k_\mathrm{t} + 4k_\mathrm{RISC}k_\mathrm{ISC} + k_\mathrm{t}^2\}^{1/2}). \tag{S1}$$

Solving for k_t leads to:

$$k_\mathrm{t} = \frac{k_\mathrm{D}^2 - k_\mathrm{D}k_\mathrm{s} - k_\mathrm{RISC}k_\mathrm{ISC}}{k_\mathrm{D} - k_\mathrm{s}}, \tag{S2}$$

which can be inserted in equation 6 to yield:

$$K_\mathrm{t}[Q] = \left(\frac{k_\mathrm{D}^2 - k_\mathrm{D}k_\mathrm{s} - k_\mathrm{RISC}k_\mathrm{ISC}}{k_\mathrm{D}^0 - k_\mathrm{D}^0 k_\mathrm{s}^0 - k_\mathrm{RISC}k_\mathrm{ISC}}\right)\left(\frac{k_\mathrm{D}^0 - k_\mathrm{s}^0}{k_\mathrm{D} - k_\mathrm{s}}\right) - 1. \tag{S3}$$

Considering the magnitudes of the different rates in Eq. S3 for organic TADF molecules ($k_D << k_s$) equation S3 can be further simplified to:

$$K_\mathrm{t}[Q] \approx \frac{k_\mathrm{D}k_\mathrm{s} + k_\mathrm{RISC}k_\mathrm{ISC}}{k_\mathrm{D}^0 k_\mathrm{s}^0 + k_\mathrm{RISC}k_\mathrm{ISC}} \frac{k_\mathrm{s}^0}{k_\mathrm{s}} - 1. \tag{S4}$$

As such, the quenching efficiency, i.e. the triplet SV constant, can not be determined only from k_D and k_D^0.

S5. Verification of equation 9

In the following, the validity of equation 9 is demonstrated using a Monte Carlo (MC) simulation as well as an analytic approach employing the rate equations of the TADF molecule-quencher system. Details on the MC simulation can be found elsewhere[1]. In the MC simulation, the diffusion length of singlet excitons was kept fixed at 18.1 nm (including singlet-triplet cycling) and the triplet exciton diffusion length was changed in the range of 0 nm to 55 nm by adjusting the transfer rates of triplet excitons between the TADF molecules. Each set of singlet and triplet diffusion length was simulated for 20 different quencher concentrations in the range of $0\,\mathrm{nm}^{-3}$ to $0.02\,\mathrm{nm}^{-3}$. By analysing the simulated PL kinetics using equation 9 the triplet diffusion length was extracted and compared to the set value of the simulation. Figure S2 demonstrates the agreement between the set and extracted value of the simulation. All simulated data is based on the rates of the TADF molecule 4TCzBN.[2]

For the second approach, the necessary quantities (k_s, χ_D) to extract the triplet and singlet diffusion length are simulated for different quencher concentrations by linearly increasing $k_{\mathrm{s/t}}^{Q}[Q]$ using the analytic solution to equation 1. The special solution for the initial conditions $c_\mathrm{s}(0) = 1$ and $c_\mathrm{t}(0) = 0$ (photoexcitation), no generation at later times and requiring that the solution is bounded for $t \to \infty$ is given by:

$$\begin{pmatrix} c_\mathrm{s} \\ c_\mathrm{t} \end{pmatrix} = -\alpha \begin{pmatrix} C_1 \\ 1 \end{pmatrix} e^{-k_\mathrm{P}t} + \alpha \begin{pmatrix} C_2 \\ 1 \end{pmatrix} e^{-k_\mathrm{D}t}, \tag{S5}$$

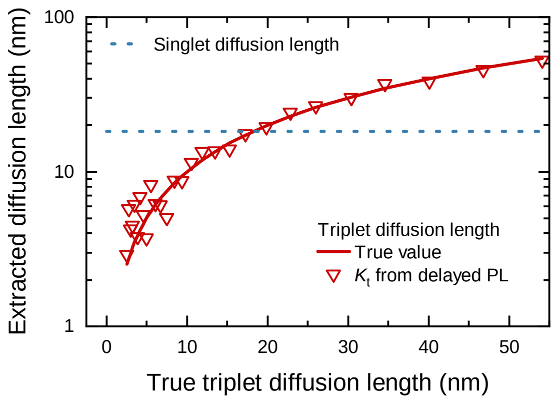

Fig. S2 Extracted diffusion length of triplet excitons using a MC simulation and SV plots based on equation S4. The diffusion length of the singlets was kept fixed at 18.1 nm throughout the simulations.

with the prompt and delayed decay rates

$$k_P, k_D = 1/2(k_s + k_t \pm \{k_s^2 - 2k_s k_t + 4k_{RISC}k_{ISC} + k_t^2\}^{1/2}), \tag{S6}$$

respectively, and $C_1 = (k_t - k_P)/k_{ISC}$, $C_2 = (k_t - k_D)/k_{ISC}$ and $\alpha = k_{ISC}/(-k_P + k_D)$.

The solution for the singlet concentration c_s is used to simulate the PL decay profiles for different quenching concentrations for singlet and triplet excitons. From these data the SV plots based on equation 5 and 9 can be constructed and are compared to the standard SV plots as described by equation 3 for three different cases, 1) singlet quenching/motion only ($K_s = 0.3$), 2) singlet and triplet quenching/motion ($K_s = 0.3$ and $K_t = 0.06$) and 3) triplet quenching/motion only ($K_t = 0.06$). Figure S3 shows the simulated PL transients for those different quenching scenarios. In case only singlet excitons are quenched, the delayed decay rate converges to the triplet decay rate k_t with increasing $[Q]$ (averaged number of singlet triplet cycles is approaching one with increasing $[Q]$).

Figure S4 depicts the SV plots of singlet and triplet excitons extracted from the PL transients shown in Figure S3. As evidenced by Figure S4 both singlet and triplet quenching are accurately described within this SV analysis.

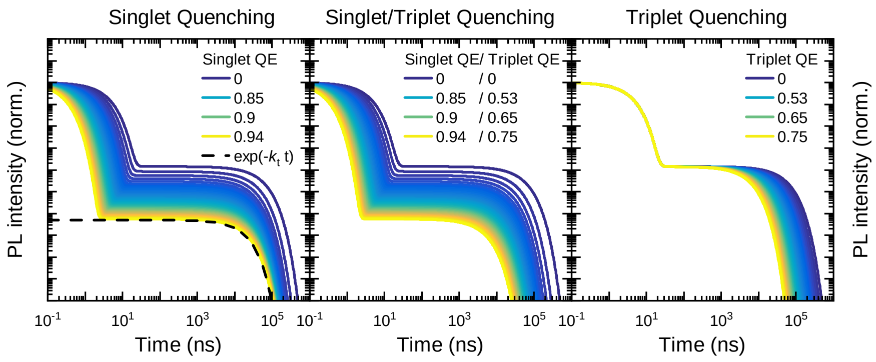

Fig. S3 Simulated PL kinetics for the molecules 4TCzBN for three different quenching scenarios. The stated numbers in the legend correspond to the singlet and triplet quenching efficiencies given by $1 - k_s^0/k_s$ and $1 - k_t^0/k_t$, respectively. The dashed line (asymptote for quenching of the delayed component) for the case of singlet quenching only corresponds to a mono exponential function with decay rate k_t.

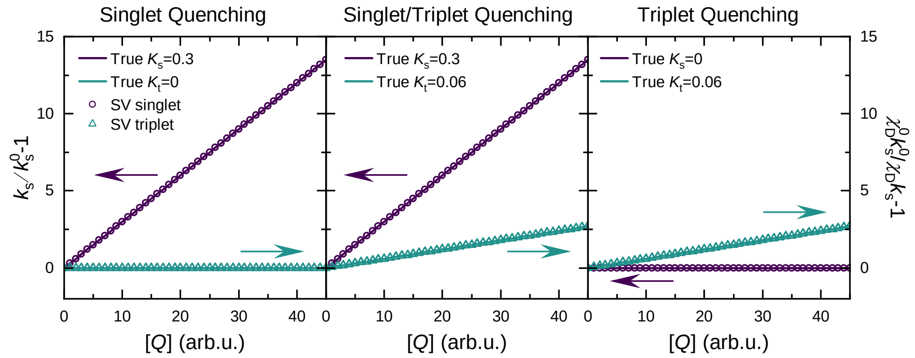

Fig. S4 Simulation of a SV plot for the three different singlet and triplet motion scenarios. The data are determined using the PL transients shown in Figure S3 together with equation 5 for singlet and equation 9 for triplet excitons.

S6. Spectroscopic data

Time-resolved PL spectra were measured with a ICCD camera (4CzIPN and 4TCzBN) and a streak camera (DiKTa) system. Details about the two setups can be found in Section S1. The collected data (Figure S5 and S6) were used to determine the prompt quenching as a function of quencher concentration based on the rate k_s and the quenching of the delayed PL fraction χ_D. The delayed decay rate of a pristine film was used to translate the determined triplet diffusion length to a triplet diffusion constant (see Section S8).

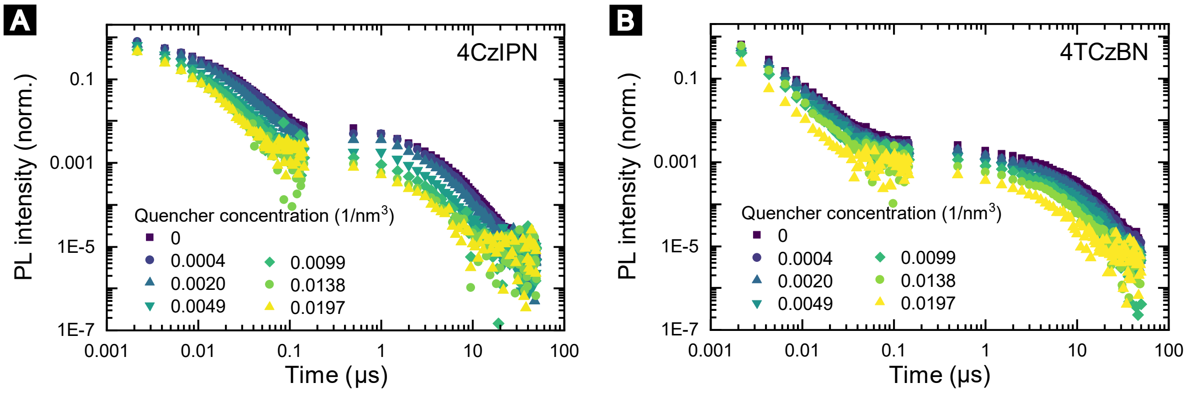

Fig. S5 PL transients of the 4CzIPN (A) and 4TCzBN (B) quenching series excited at 355 nm and recorded with an ICCD camera.

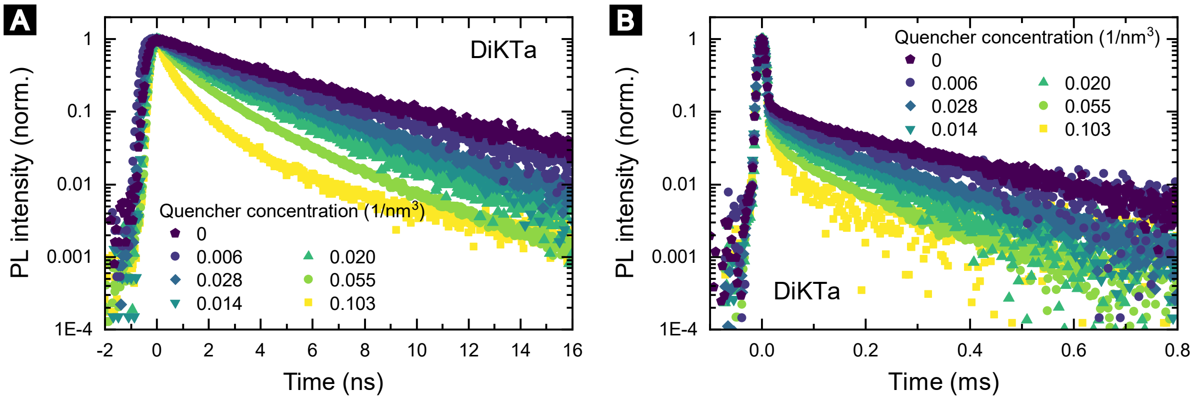

Fig. S6 PL transients of the DiKTa quenching series excited at 343 nm. (A) PL transients extracted from a streak image with a time base of 20 ns. (B) PL transients taken from a streak image with a time base of 1 ms.

Q. con. (nm^{-3})	4CzIPN k_s (ns^{-1})	4CzIPN χ_D	4CzIPN k_D (ns^{-1})	4TCzBN k_s (ns^{-1})	4TCzBN χ_D	4TCzBN k_D (ns^{-1})
0	0.08	0.36	2.2×10^{-4}	0.31	0.55	4.5×10^{-4}
0.006	0.09	0.33		0.33	0.52	
0.028	0.11	0.32		0.42	0.51	
0.014	0.17	0.22		0.52	0.47	
0.020	0.22	0.15		0.66	0.34	
0.055	0.27	0.14		0.86	0.29	
0.103	0.33	0.11		0.98	0.23	

Table S1 Extracted parameter from the PL transients shown in Figure S5. The decay rates k_s and k_D are taken from mono exponential fits of the prompt and delayed PL transient, respectively. χ_D is determined from individual integration of the prompt ($0\,ns$ to $150\,ns$) and delayed ($0.3\,\mu s$ to $100\,\mu s$) PL decay transient.

Q. con. (nm^{-3})	DiKTa k_s (ns^{-1})	DiKTa χ_D	DiKTa k_D (ns^{-1})
0	0.23	0.73	4.3×10^{-6}
0.006	0.29	0.68	
0.028	0.37	0.45	
0.014	0.41	0.58	
0.020	0.49	0.52	
0.055	0.70	0.34	
0.103	1.23	0.20	

Table S2 Extracted parameter from the PL transients shown in Figure S6. The singlet decay rates k_s for the different quencher concentrations are determined from mono exponential fits of the prompt PL transients shown in Figure S6 (A). χ_D determined from separate integration of the prompt and delayed component of the PL transient shown in Figure S6 (B). The delayed decay rate k_D was determined from a mono exponential fit of the delayed PL transient.

S7. Calculation of the diffusion length and diffusion constant

The singlet and triplet exciton diffusion lengths for one cycle through the respective state have been determined from the SV plots employing the Smoluchowski equation, as already laid out in the main manuscript. For singlet excitons this diffusion length can directly be translated to a diffusion constant by using the prompt decay rate, according to:

$$D = \frac{1}{6} k l^2. \tag{S7}$$

For triplet excitons, the determined diffusion length were first multiplied by a factor $\langle \sqrt{n_\mathrm{S}} \rangle = \sqrt{\frac{1}{1-\chi_\mathrm{D}}}$ to determine the diffusion length for all cycles. In a second step, equation S7 was used together with the delayed decay rate to determine the diffusion constant. It should be noted that the used definition for the total diffusion length of triplet excitons does not include excitons that did not cycle once through the triplet state, i.e. singlet excitons that undergo radiative decay directly after photoexcitation are not accounted for (with a triplet diffusion length of 0 nm).

S8. Details on the density functional theory (DFT) simulations

Atomistic film morphologies with dimensions of at least 16x16x16 nm^3 were generated using Deposit[3], a molecular modeling tool which simulates physical vapor deposition using force field methods. During deposition, bonds and bond-angles were kept rigid and only rotation around single-bond dihedrals was allowed. The intramolecular interaction was computed with an internal non-bonded force field and a customized dihedral force field. The dihedral force field was derived by rotating single groups step wise and computing the energy profile around 360° at 18 steps with DFT (BP, def2-SVP). Non-bonded force field parameters were then fitted to full-DFT energies for a set of training molecules in random configurations, taking into account the formerly derived DFT-based dihedral energy profiles. The Quantum Patch approach[4–6] was used to estimate partial charges for each molecule in the morphology from semi-empirical DFTB+[7] calculations. These partial charges were used to represent the molecular environment in DFT calculations of pairs of the ten central emitter molecules to obtain charge and Dexter transfer couplings. Charge transfer couplings were obtained via the direct coupling scheme,

$$V_{if}^{\mathrm{charge}} = \frac{\langle i|F|f\rangle - \frac{1}{2}\left(\langle i|F|i\rangle + \langle f|F|f\rangle\right)\langle i|S|f\rangle}{1-\langle i|S|f\rangle^2}, \tag{S8}$$

where i and f denote initial (prior to transfer) and final (after transfer) states, respectively, F is the Fock matrix and S the overlap matrix, and we only take into account the relevant orbitals (HOMO for hole transfer, LUMO for electron transfer), while Dexter couplings were approximated as

$$V_{if}^{\mathrm{Dexter}} \approx \sum_{n,m} \langle \mathrm{HOMO}, A|n\rangle \langle \mathrm{HOMO}, B|n\rangle \langle n|H|m\rangle \langle m|\mathrm{LUMO}, A\rangle \langle m|\mathrm{LUMO}, B\rangle. \tag{S9}$$

Hopping rates were calculated according to Marcus theory[8],

$$k = \frac{2\pi}{\hbar} |V_{if}|^2 \sqrt{\frac{1}{4\pi k_\mathrm{B} T \lambda}} \exp - \frac{(\lambda + \Delta E_{if})^2}{4\lambda k_\mathrm{B} T}, \tag{S10}$$

where ΔE_{if} is the energy difference between the initial and final state, $T = 300\,\mathrm{K}$ the temperature, and λ the reorganization energy, which was estimated from DFT calculations of the emitter molecules in vacuum using Nelson's four-point procedure[9]. Here, we neglect energetic disorder and therefore set $\Delta E_{if} = 0$.

Unless otherwise specified, DFT calculations were carried out using the B3LYP exchange-correlation functional and def2-SVP basis set as implemented in TURBOMOLE[10].

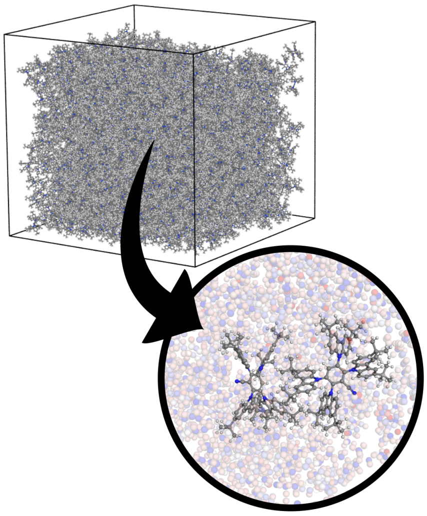

Fig. S7 Schematic of the simulation workflow. First morphologies are generated emulating physical vapor deposition. For the calculation of the triplet transfer rates molecule pairs are randomly selected and the transfer rate is computed in accordance with Marcus theory. All other molecules are estimated as partial charges and represent the molecular environment in the DFT calculations.

S9. Results of the DFT simulations

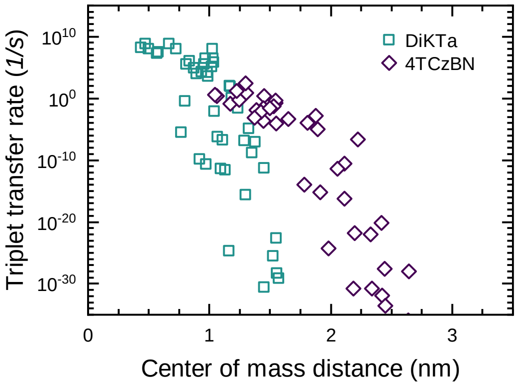

Fig. S8 Determined Dexter transfer rates for the molecules DiKTa and 4TCzBN in pure TADF morphology. The rates are calculated using a DFT simulation detailed in Section S8.

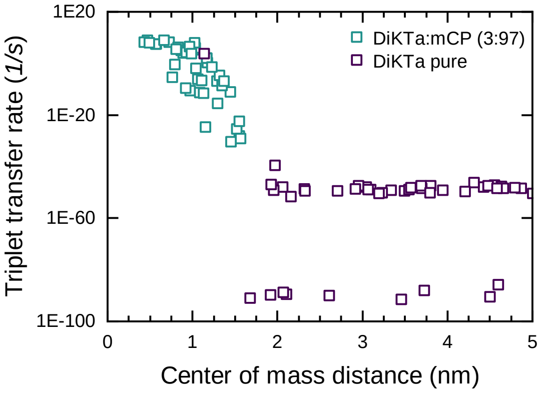

Fig. S9 Comparison of the triplet transfer rates of a pure DiKTa film and a 3:97 (wt.) mixture of DiKTa and mCP. Note, that due to numeric artifacts (rounding of small numbers) the rates for the mixed morphology are mainly centered around $1 \times 10^{-50}\,\mathrm{s}^{-1}$ and $1 \times 10^{-90}\,\mathrm{s}^{-1}$ but the determined values can be seen as upper bounds.

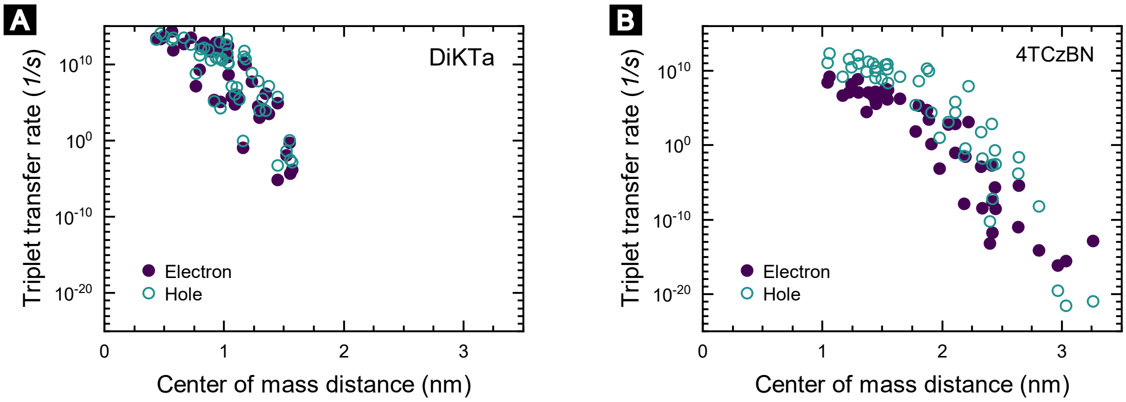

Fig. S10 Determined electron and hole transfer rates of DiKTa (A) and 4TCzBN (B).

Notes and references

1 M. Jakoby, B. S. Richards, U. Lemmer and I. A. Howard, *Phys. Rev. B*, 2019, **100**, 045303.

2 D. Zhang, M. Cai, Y. Zhang, D. Zhang and L. Duan, *Mater. Horiz.*, 2016, **3**, 145–151.

3 T. Neumann, D. Danilov, C. Lennartz and W. Wenzel, *J. Comput. Chem.*, 2013, **34**, 2716–2725.

4 P. Friederich, F. Symalla, V. Meded, T. Neumann and W. Wenzel, *J. Chem. Theory Comput.*, 2014, **10**, 3720–3725.

5 P. Friederich, V. Meded, F. Symalla, M. Elstner and W. Wenzel, *J. Chem. Theory Comput.*, 2015, **11**, 560–567.

6 P. Friederich, R. Coehoorn and W. Wenzel, *Chem. Mater.*, 2017, **29**, 9528–9535.

7 M. Elstner, D. Porezag, G. Jungnickel, J. Elsner, M. Haugk, T. Frauenheim, S. Suhai and G. Seifert, *Phys. Rev. B*, 1998, **58**, 7260–7268.

8 R. A. Marcus, *Rev. Mod. Phys.*, 1993, **65**, 599–610.

9 J.-L. Brédas, D. Beljonne, V. Coropceanu and J. Cornil, *Chem. Rev.*, 2004, **104**, 4971–5004.

10 R. Ahlrichs, M. Bär, M. Häser, H. Horn and C. Kölmel, *Chem. Phys. Lett.*, 1989, **162**, 165–169.
